# Regulation of SUMOylation on RNA metabolism in cancers

**DOI:** 10.3389/fmolb.2023.1137215

**Published:** 2023-02-24

**Authors:** Yingting Cao, Caihu Huang, Xian Zhao, Jianxiu Yu

**Affiliations:** Department of Biochemistry and Molecular Cell Biology and Shanghai Key Laboratory of Tumor Microenvironment and Inflammation, Shanghai Jiao Tong University School of Medicine, Shanghai, China

**Keywords:** sumoylation, mRNA transcription, mRNA processing, RNA editing, miRNA biogenesis, cancer

## Abstract

Post-translational modifications of proteins play very important roles in regulating RNA metabolism and affect many biological pathways. Here we mainly summarize the crucial functions of small ubiquitin-like modifier (SUMO) modification in RNA metabolism including transcription, splicing, tailing, stability and modification, as well as its impact on the biogenesis and function of microRNA (miRNA) in particular. This review also highlights the current knowledge about SUMOylation regulation in RNA metabolism involved in many cellular processes such as cell proliferation and apoptosis, which is closely related to tumorigenesis and cancer progression.

## Introduction

Small ubiquitin-like modifier (SUMO), a member of the ubiquitin-like post-translational modifiers are well conserved in eukaryotes (Wilkinson and Henley, 2010; Wang and Yu, 2021). Up to now, mass spectrometry and bioinformatics analysis provide proteomic evidence for SUMOylation of 3,617 proteins at 7,327 SUMOylation sites (Hendriks and Vertegaal, 2016). Although SUMO targets are distributed in various distinct sub-cellular structures, nucleoproteins still account for the majority. SUMOylation has an important regulatory role for most nuclear processes, including RNA transcription, primary RNA processing, nucleocytoplasmic transport, cell cycle progression, nuclear body formation and protein stabilization (Hendriks and Vertegaal, 2016). SUMOylation affects cancer progression by regulating tumorigenesis, proliferation, transformation, migration, invasion, senescence, inflammation, angiogenesis, etc. Therefore, SUMO-related enzymes that involve in tumorigenesis can be used as targets for drug development to treat human cancers.

SUMOylation is a reversible process of adding SUMO to substrate proteins at specific lysine residues as a post-translational modification (PTM) (Wang and Yu, 2021). In mammalian cells, SUMO proteins are conjugated to over thousands proteins. SUMO is conserved from yeast to mammalian cells. Thus far, there are five SUMO isoforms with different sequences in mammals: SUMO1, 2, 3, 4 (Han et al., 2018) and 5 (Liang et al., 2016). SUMO1, SUMO2 and SUMO3 are widely expressed in humans, while SUMO4 and SUMO5 are only found in specific tissues or organs (Guo et al., 2004). SUMO2 and SUMO3 share about 97% identity in humans and are often known as SUMO2/3. SUMO1 only have 53% identity with SUMO2/3. SUMO4 has an 86% amino acid homology with SUMO2, containing a unique proline-90 residue may prevent the maturation and disrupting conjugation to substrates (Owerbach et al., 2005). SUMO5 is reported as a novel SUMO that highly homologous to SUMO1, and poly-SUMO5 conjugation on promyelocytic leukaemia protein (PML) results in the recruitment of proteins to form PML nuclear bodies (PML NBs) (Liang et al., 2016). So far, the research on SUMO4 and SUMO5 is limited.

**FIGURE 1:**
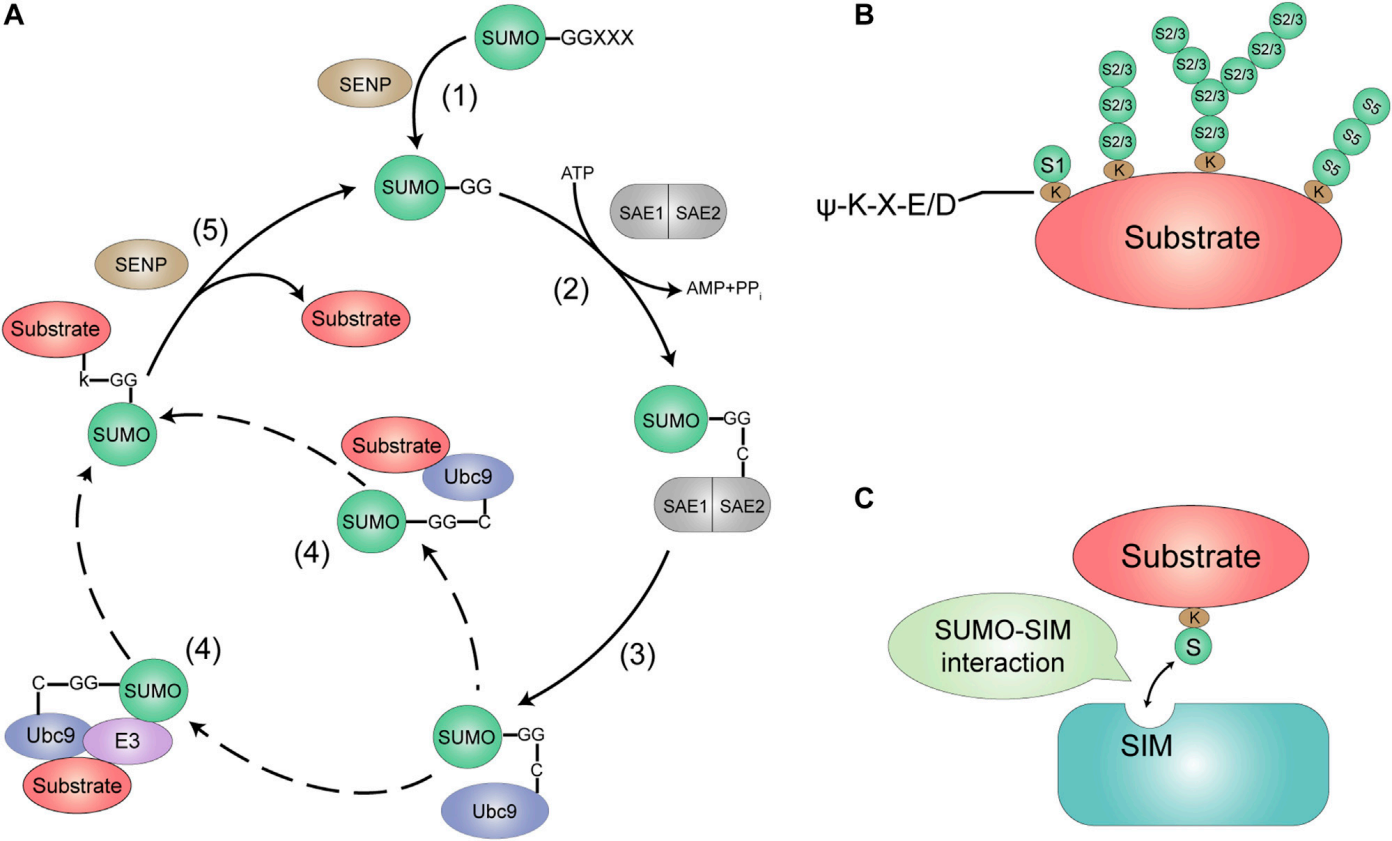
The SUMOylation system. **(A)** SUMO is encoded as an inactive precursor that is cleaved by members of the SENP family to expose the C-terminal diglycine motif (1). This mature form of SUMO is then activated by forming an ATP-dependent thioesler bond with the active site of the Et enzyme (a heterodimer of SAE1 and SAE2) (2). The activated SUMO is then delivered to the active site cysteine of the E2-conjugating enzyme Ubc9. Which then catalyzes the transfer of SUMO to the target protein, either alone or with the help of a SUMO E3 ligase (3, 4). SUMOylated sub-strates show phenotypic differences from the unmodified form. DeSUMOylation is mediated by the SENP protease family (5). This process releases unmodified target protein (not shown) and mature SUMO. which can then be used for further binding to the target protein. **(B)** SUMO modifications most commonly target proteins with the consensus motif w-K-X-FJD. to is a hydrophobic amino acid. K is the target Lys. X is any amino acid, and DIE is Asp or Glu. SUM01 modifies substrates into monomers. While SUMO2/315 modifies substrates in poly•SUMO chains. **(C)** Proteins contain-ing SIMs mediate non-covalent interactions with SUMO.

Similar to the biochemical process of ubiquitination, SUMO attaches to the Lys K) residues of target proteins *via* an isopeptide bond between its C-terminal glycine G) and the ε-NH2 group of a lysine residue in the target protein (Gill, 2004). SUMOylation is a multi-step enzymatic process, including activation, conjugation and ligation (Figure 1A). The first step of SUMO conjugation pathway is the maturation of SUMOs, whose COOH termini is cleaved by Sentrin-specific proteases (SENPs) to expose the di-glycine (GG) residues required for conjugation (Hay, 2007). Second, mature SUMOs are then activated through an ATP-dependent way by a heterodimeric SUMO E1-activating enzyme, SAE1/2, which is a heterodimer composed of Aos1/SAE1 and Uba2/SAE2 with a size of 110 kDa (Desterro et al., 1999). The SUMOs exclusively interact with the SAE2 subunit, and their C-terminal GG carboxyl group is activated by linking with cysteine C) residue of SAE2 through a thioester bond. Third, the activated SUMO is then transferred to a SUMO conjugating enzyme E2, Ubc9. Fourth, SUMO transferring of Ubc9-SUMO has two ways, by which Ubc9 catalyzes an isopeptide bond between the SUMO terminal GG and the K side chain of the substrate proteins by direct interacting with or without a E3 ligase enzyme. The final step, SUMOylation is reversed by the family of SUMO-specific proteases SENPs that break isopeptide bonds to remove SUMO from the substrate, recycling SUMO molecules (Yeh, 2009; Chang and Yeh, 2020).

In general, the most common target proteins of SUMO modification are to have a common motif ψ-K-X-E/D (ψ is a hydrophobic amino acid, K is the target Lys, X is any amino acid, D/E is amino acid Asp/Glu), but there are also some proteins without classical motifs that have been more reported recently (Chang and Yeh, 2020). In usual, SUMO1 modifies the substrate proteins by one monomer, while SUMO2/3 can modify by poly-SUMO to form multiple SUMO chains. At present there is no evidence that SUMO4 is conjugated to K of substrate protein, but SUMO5 seems to be able to form a poly-SUMO chain (Figure 1B). Reader proteins can recognize and bind to SUMOylated proteins through their SUMO interaction motifs (SIMs). (Martin et al., 2007) (Figure 1C). Functionally, SUMO is covalently coupled to a large number of proteins to modulate their enzymatic activity, subcellular localization or their protein interactions. SUMOylation may also join with ubiquitin to degrade proteins through SUMO-targeting ubiquitin ligase (STUbL) (Hickey et al., 2012).

Importantly, SUMOylation plays an important role in RNA metabolism, including messenger RNA (mRNA) and non-coding RNA (ncRNA). Abnormal RNA metabolism caused by SUMOylation is often the main reason affecting the occurrence and development of tumors. On the one hand, mRNA metabolism regulated by SUMOylation involves in transcription, processing (e.g., capping, splicing and polyadenylation), mRNA translation and quality control. On the other hand, SUMOylation of regulation on ncRNA metabolism is mostly and broadly focused on microRNAs (miRNAs). In addition, researches on cellular processes compartmentalized by membrane-less organelles (MLOs) have become a hotspot. MLOs typically contain disordered proteins and RNAs by liquid-liquid phase separation (LLPS) (Alberti and Hyman, 2021). MLOs for molecular condensates in the nucleus include nucleoli (Tartakoff et al., 2022), paraspeckles (Yamazaki et al., 2021), nuclear speckles (NS) (Spector and Lamond, 2011), Cajal bodies (Nizami et al., 2010), PML NBs (Lallemand-Breitenbach and de The, 2018) and nuclear stress bodies (nSBs) (Biamonti and Vourc’h, 2010), and in the cytoplasm there are P-bodies and stress granules (SGs) (Ivanov et al., 2019). They function as RNA and protein quality control centers and are highly sensitive to cellular stress, including proteotoxic stress (Advani and Ivanov, 2019; Hirose et al., 2022). Growing evidence shows that SUMOylation is critically involved in regulating both the assembly and disassembly of MLOs, and thus therefore regulates RNA splicing, processing and RNA stability (Hofweber and Dormann, 2019).

This review provides insights into the role of SUMOylation in regulating RNA metabolism in the pathophysiology of diseases, especially cancers. We summarize the regulation of RNA metabolism by SUMOylation from the following aspects: 1) RNA transcription; 2) mRNA processing; 3) RNA stability; 4) RNA editing and RNA modification; 5) Non-coding RNA metabolism.

## RNA transcription

SUMOylation plays an important role in transcriptional regulation *via* SUMO conjugating to histones, histone-modifying enzymes, transcription factors, transcriptional co-regulators and transcriptional machinery-related proteins. The mechanisms underlying SUMO-mediated transcriptional regulation includes alterations in subcellular localization, DNA binding, protein-protein interaction, stability and enzymatic activity (Chymkowitch et al., 2015).

**FIGURE 2:**
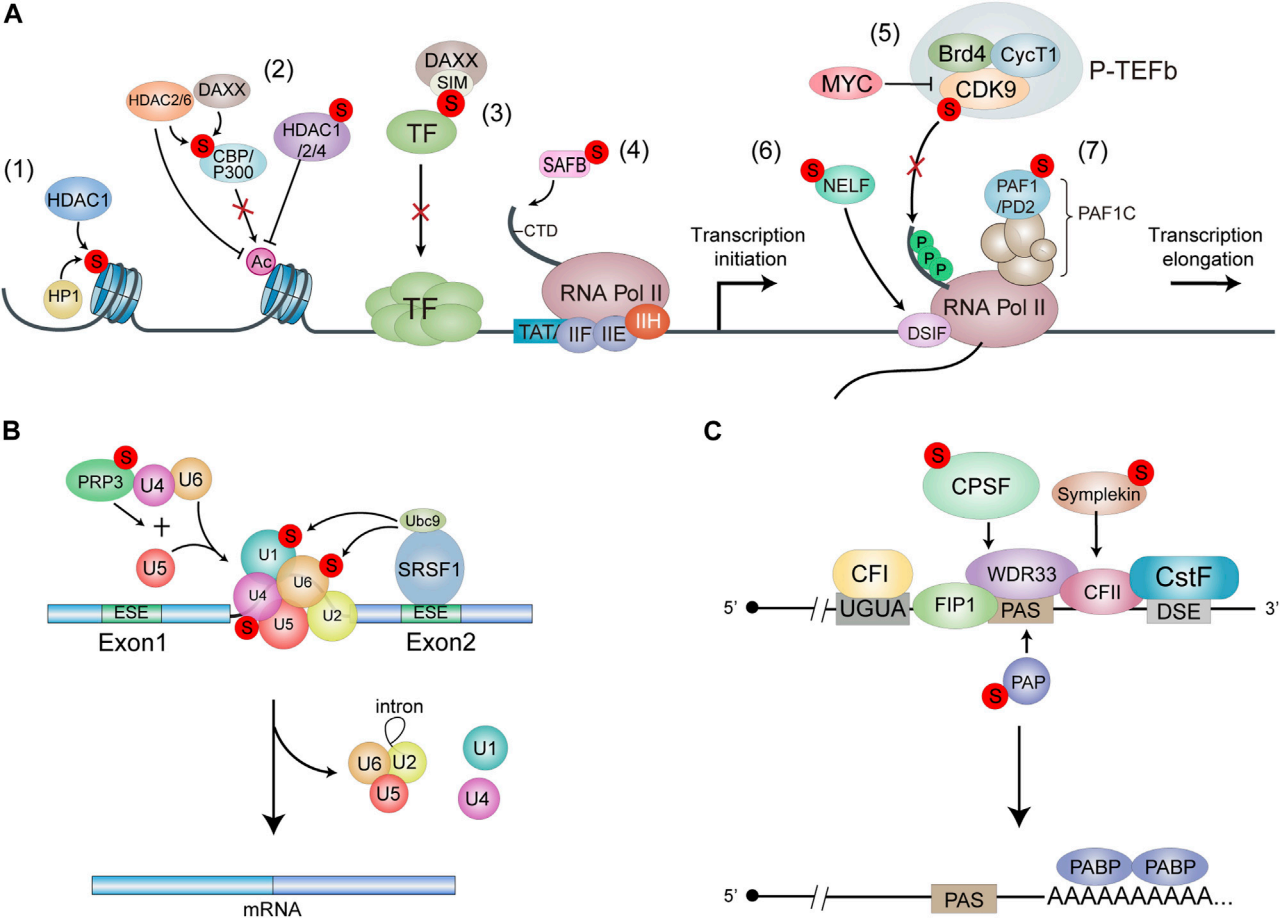
Models for the functions of SUMOylation in RNA transcription and processing. **(A)** SUMOylation leads to transcnptional repression. Histone SUMOylation mediates transcriptional repression through recruitment of HDAC1 and HP1 (1). SUMOylation of p300 and CBP recruits HDAC6 and DAXX/HDAC2 respechvehi, leading to SUMO-dependent transcnptional repression. SUMOylation of liADC1/2/4 promotes the deacetylase activity to repress transcription (2). The SIM domain of DAXX provides a molecular explanation for the interaction between DAXX and SUMO-mod-ified transcription factors, resulting In transcriptional repression (3) SUMOylation of SAFE enhances its binding with RNAPII, thereby promoting gene transoiption (4). SUMO and MYC antagonistically control global gene expression through regulating COK9 SUMOylation. P-TEFb formation and tran-scriptional elongation. SUMOylation of CDK9 blocks its interaction with Cyclin Ti. Thereby inhibiting the formation of active P-TEFb complex (5). NELF and OSIF bind to the potymerase in a manner that restricts Pol II mobility and impairs further RNA elongation. SUMOylation regulates the recruitment of NELF to promoters upon stress to drive transcriptional downregulabon (6). SUMOytabon of PAF1/PD2 facilitates its interaction with PML proteins (7). **(B)** SRSF1. Bound to the exonic splicing enhancer (ESE). Interacts with Ubc9 to regulate the SUMOylation of spliceosome protein components. Thuieby increasing splicing efficiency. PRP3 acts as a component of the 114/U6 cli-snRNP. and Its SUMOylatlon Promotes U41U6/1.15 tri-snRNP formation to effect splicing. **(C)** SUMOylation of CPSF. PAP and Sim-pleton affects the assembly and activity of pre-mRNA 3’complex.

Histones undergo many modifications, including acetylation, methylation, phosphorylation, ubiquitination, glycosylation, ADP ribosylation and SUMOylation, some of which play key roles in the regulation of chromatin structure and functions. Most histones, including H1, H2A, H2B, H3, H4 and H2A.X are SUMOylated (Table 1). SUMOylation of H4 leads to chromatin compaction and transcriptional repression through the recruitment of histone deacetylase 1 (HDAC1) and heterochromatin protein 1 (HP1) (Shiio and Eisenman, 2003; Ryu and Hochstrasser, 2021) (Figure 2A; Figure 1). However, SUMOylation of H4K12 has also been reported to inhibit chromatin compaction (Dhall et al., 2014). Moreover, SUMOylation of linker histone H1 drives chromatin condensation and restriction of embryonic cell fate identity (Dhall et al., 2014). The transcriptional repression mechanisms of SUMOylation at other histones are not very clear.

**TABLE 1:** SUMOylation of histones, HATs and HDACs.

Type	Proteins	SUMO 1/2/3	Sites	Function	References
Histones	H2A	S1/3		Transcriptional repression or chromatin compaction	Shiio and Eisenman (2003), Chen et al. (2013)
	H2B	S1/3			(Shiio and Eisenman, 2003) (Ryu et al., 2020)
	H3	S1/3	K18		Shiio and Eisenman (2003)
	H4	S1/3	K12	Inhibits chromatin compaction	Shiio and Eisenman (2003), Chen et al. (2013), Dhall et al. (2014), Dhall et al. (2017)
	H2A.X	S1	K5, K9, K13, K15, K118, K119, K127, K133, K134		Chen et al. (2013)
	H1	S1		Chromatin condensation	Matafora et al. (2009)
HDACs	HDAC1	S1/2	K444, K476	Enhances histone deacetylase activity	David et al. (2002), Cheng et al. (2004), Citro et al. (2013)
	HDAC4	S1/2	K559	Enhances histone deacetylase activity	Kirsh et al. (2002)
	HDAC2	S1	K462	Recruits to promoters	Brandl et al. (2012)
HATs	EP300	S1/2/3	CRD Domain	Recruits HDAC6	Girdwood et al. (2003), Park et al. (2017)
	CBP	S1	K999, K1034, K1057	Recruits DAXX and HDAC2	Kuo et al. (2005), Park et al. (2017)

As known, the acetylation level of histone is positively correlated with gene activation. Histone acetylation is catalyzed by histone acetyltransferases (HATs) such as CREB-binding protein (CBP) and p300 while it is removed by histone deacetylase (HDACs). CBP and p300 are co-regulators of many sequence-specific DNA binding proteins in response to a variety of signaling (Yu et al., 2004), and are regarded as key drivers of tumorigenesis (Hogg et al., 2021). Both p300 and CBP contain a cell cycle regulatory domain 1 (CRD1), which is a strong transcriptional repression domain. It has been reported that SUMOylation of p300 CDR1 domain mediates transcriptional repression by promoting the recruitment of HDAC6 (Girdwood et al., 2003) (Figure 2A–2). Similarly, SUMOylation of CBP negatively modulates transcriptional activity by recruiting Death domain-associated protein (DAXX) and then HDAC2 (Kuo et al., 2005; Park et al., 2017).

On the other hand, HDACs reverse chromatin acetylation by removing acetyl groups and alter the transcription of oncogenes and tumor suppressor genes governing cancer initiation and progression (Li and Seto, 2016). HDACs family members such as HDAC1, HDAC2 and HDAC4 can also be SUMOylated. SUMOylation of HDAC1/HDAC4 enhances their histone deacetylase activity, leading to the transcriptional repression (David et al., 2002; Kirsh et al., 2002). Moreover, SUMOylation of HADC4 can enhance its deacetylation of p53, thus blocking the recruitment of p53 into the promoter, which modulates the apoptotic response to genotoxic stress in cancer cells (Brandl et al., 2012). In addition to enhancing the deacetylase activity, SUMOylation of HDAC2 also increases its binding to the Elk-1 regulated promoters, which directly leads to a decrease of histone acetylation and hence transcriptional repression at Elk-1 target genes (Yang and Sharrocks, 2004) (Figure 2A). HDACs are abnormally high in a variety of cancers, which govern a wide array of biological processes including cancer initiation and progression, thus the development of HDACs-targeted imaging probes for cancer detection and targeting HDACs for cancer therapy have become a hotspot in epigenetic research (West and Johnstone, 2014; Tang et al., 2022). Since SUMOylation of HDACs plays an important role in the regulation of HDACs activity in cancer cells, it is worthwhile to design some small chemical molecules or other specific drugs to interfere with the SUMO modification of HDACs in cancers.

**FIGURE 3:**
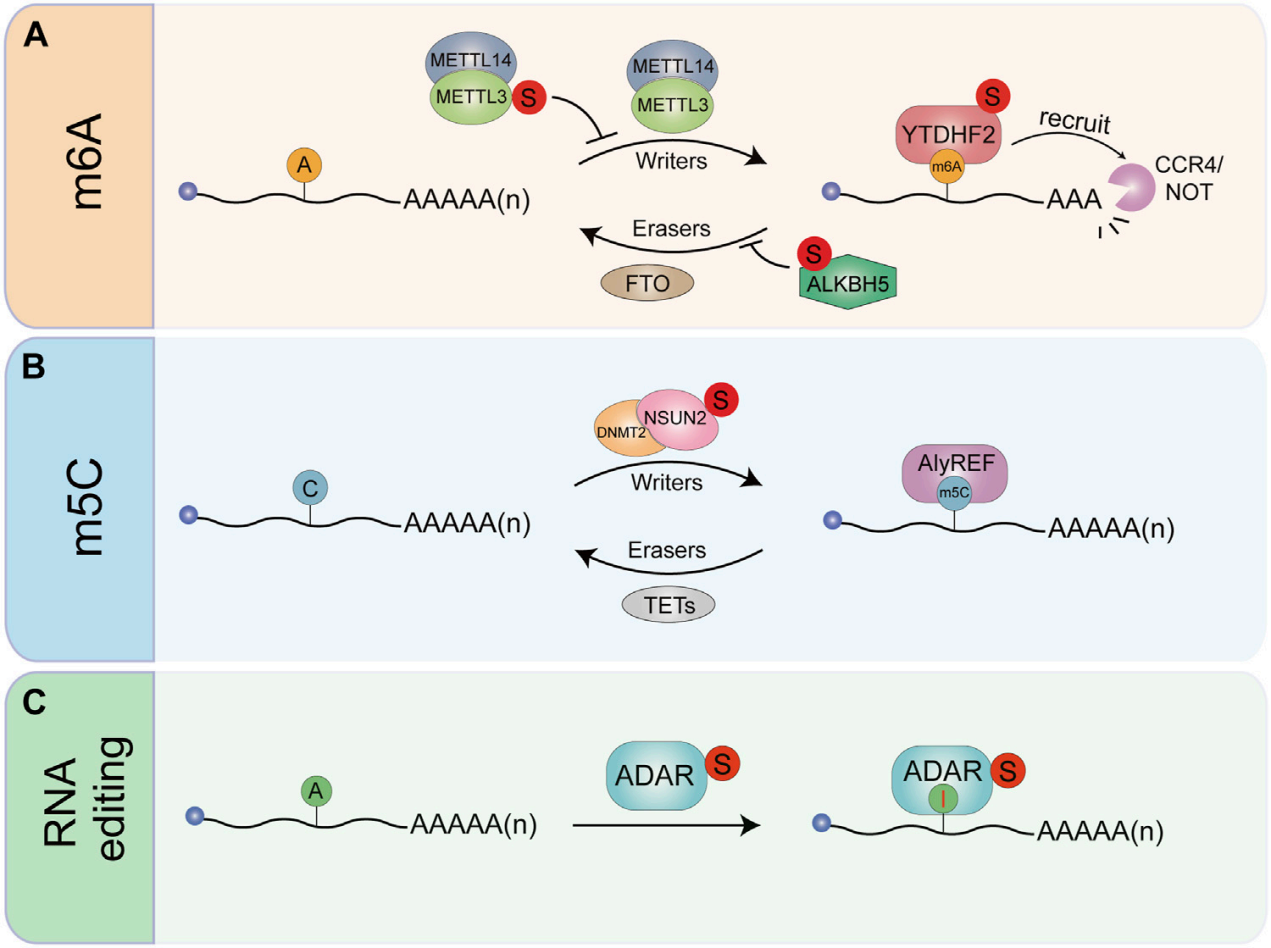
The effects of SUMOylation on RNA modification and RNA editing. **(A)** The N6-methylad-enosine writer METTL3. Reader YTHDF2 and eraser ALKBHS can be modified by SUMO. SUMOyIa-lion of METTL3 does not affect its stability. Subcellular localization. Or Interaction with MET11141 W-TAP. but significantly inhibits METTL3 m6A methyltransferase activity. YTHDF2 can be modified by SUMO1 under hypoxia. Enhancing its binding affinity to m6A-RNAs. Resulting in dysregulation of gene expression. ALKBHS SUMOylation loads to inhibition of ALKBHS m6A demethylase activity. Thereby increasing global mRNA m6A levels. **(B)** SUMOylation stabilizes m5C writer NSUN2 and mediates its transport into the nucleus. **(C)** The RNA editing enzyme ADAR1. Which converts ade-nosine to inosine, can be SUMOylated thereby inhibiting its RNA editing activity.

A wide range of transcription factors and co-transcriptional regulators have been reported as SUMOylated proteins. In most cases, SUMOylation is usually associated with transcriptional repression (Rouviere et al., 2013). Here, we list transcription regulators, especially those related to cancer, which can be modified by SUMOs to modulate transcriptional activation activity (Table 2). Remarkably, DAXX regulates gene expression as a transcriptional co-repressor or co-activator by interacting with diverse core histones, chromatin-associated proteins, transcription factors and epigenetic regulators (Mahmud and Liao, 2019). DAXX-regulated genes are important effectors in cell death, survival and tumorigenesis (Mahmud and Liao, 2019), so the expression level of DAXX is increased in several cancer types including prostate (Tsourlakis et al., 2013), ovarian (Pan et al., 2013) and gastric cancer (Xu et al., 2017). DAXX can be modified by SUMO1 increasing recruitment of DAXX to PML-NBs (Jang et al., 2002), which induces cancer cell apoptosis (Takahashi et al., 2004). Moreover, the conserved SIMs of DAXX provide a molecular explanation for the interactions of DAXX with SUMO-modified proteins, leading to transcriptional activation or repression (Figure 2A; Figure 3). For examples, DAXX can interact SUMO-modified androgen receptor (AR) and glucocorticoid receptor (GR) to repress their transcriptional activities by inhibiting their DNA binding (Lin et al., 2004; Lin et al., 2006). By binding with SUMOylated-SMAD4, DAXX inhibits the tumor suppressive effect of transforming growth factor-β (TGF-β) signaling mediated by SMAD4 (Chang et al., 2005), which affects the growth arrest and apoptosis of cancer cells (Zhao et al., 2018).

**TABLE 2:** SUMOylation of transcription regulators.

Genes	Function in transcription	Protein roles in cancer	SUMO effects on transcription	Refs
EGR1	Transcription factor	An effect either as a growth promoter or as a tumor suppressor	Promotes PTEN transcription	Yu et al. (2004)
PAX6	Transcription factor	Important in cancer cell proliferation and tumor progression	Transcription activation; enhances the DNA-binding ability of p32 Pax-6	Yan et al. (2010)
FOXM1	Transcription factor	Oncogene; regulates the expression of target genes	Transcription activation; inhibits the negative regulatory domain of FOXM1	Schimmel et al. (2014)
P53	Transcription factor	Tumor suppressor; induces growth arrest or apoptosis	Transcription activation	Gostissa et al. (1999), Rodriguez et al. (1999), Chauhan et al. (2021)
ESR1	Nuclear hormone receptor	Essential for the normal development of the mammary gland and the tumorigenesis and progression of breast cancer	Transcription activation	Sentis et al. (2005)
E2F1	Transcription factor	Mediate cell proliferation and EZH2 overexpression-mediated cancer progression	Enhances EZH2 transcription; regulates E2F1 binding to the EZH2 promoter	Du et al. (2020)
HSF1/HSF2	Transcription factor	Over expression of HSFs associates with drug resistance and poor clinical outcomes in various malignancies	Transcription activation; increases HSF2 DNA binding activity	Goodson et al. (2001), Hong et al. (2001)
SP3	Transcription factor	Inductor of apoptosis and marker of tumor aggressiveness	Transcription repression; SP3 cannot act as a target for SUMO modification when bound to DNA.	Sapetschnig et al. (2002)
TFAP2C	Transcription factor	Links to the etiology of human breast cancer	Transcription repression	Eloranta and Hurst (2002)
CEBPA/CEBPB/CEBPE	Transcription factor	Regulates of cell growth and differentiation	Transcription repression	Kim et al. (2002)
GATA2	Transcription factor	Hematopoietic and cardiovascular development	Transcription repression	Chun et al. (2003)
SRF	Transcription factor	Controls the expression of genes regulating the cytoskeleton during development, morphogenesis and cell migration	Transcription repression	Matsuzaki et al. (2003)
PLAG1	Transcription factor	Oncoprotein; provides anti-anoikis and pro-metastatic signals in LKB1-deficient lung cancer	Transcription repression	Astrom et al. (2000), Van Dyck et al. (2004)
ELK1	Transcription factor	Enhances cancer Progression	Transcription repression	Yang et al. (2003)
MYB	Transcriptional coactivator	Controls proliferation and differentiation of hematopoietic progenitor cells	Transcription repression; increases its proteolytic stability	Bies et al. (2002)
DDX5	Transcriptional coactivator	Transcriptional coactivator of the tumor suppressor p53 and ESRRA	Transcription repression; enhances its interaction with HDAC1	Jacobs et al. (2007)
HIF1A	Transcriptional coactivator	Essential for embryonic vascularization, tumor angiogenesis and pathophysiology of ischemic disease	Transcription repression	Berta et al. (2007)
IRF1	Transcriptional activator	Tumor suppressor; antagonism of tumor cell growth	Transcription repression	Nakagawa and Yokosawa (2002)
SMAD4	Translational corepressor	Tumor suppressor	Transcription repression; enhances the stability of SMAD4; enhances its interaction with DAXX; activates TGF-β signaling	Lin et al. (2003a), Lee et al. (2003), Long et al. (2004), Chang et al. (2005), Liu K et al. (2020)
SMAD3	Translational corepressor	Tumor suppressor	Transcription repression; influences its DNA-binding activity; stimulates its export from the nucleus	Imoto et al. (2008)
CTBP	Transcription corepressor	Repressor of Wnt target gene transcription	Transcription repression; important for nuclear localization	Lin et al. (2003b)
MTA1	Transcriptional coregulator	Important in tumorigenesis, tumor invasion and metastasis	Transcription repression; recruits HDAC2 onto the PS2 promoter	Cong et al. (2011)
PGR	Progesterone receptors	Mediates proliferation during breast development and contributes to breast cancer progression	Transcription repression	Daniel et al. (2007)
ESR2	Nuclear hormone receptor	Anti-oncogene	Transcription repression; stabilizes ESR2; modulates ESR2 chromatin interaction	Picard et al. (2012)
IKBA	NF-kappa-B/REL complexes	Inhibits the activity of dimeric NF-κB complexes	Transcription repression	Desterro et al. (1998)
			Resistant degradation	
AR	Transcription factor	Enhances androgen-dependent proliferation of prostate cancer cells	Activation/Repression	Poukka et al. (2000), Zheng et al. (2006), Bawa-Khalfe et al. (2007)
SP1	Transcription factor	Tumor suppressor	Activation/Repression; SUMO1 and SUMO2 exert opposing effects on Sp1 stability	Gong et al. (2014)

Although SUMOylation of most transcription factors and transcriptional co-regulators plays a negative role in transcription, it also activates several transcription factors, such as tumor suppressor p53 (Gostissa et al., 1999; Rodriguez et al., 1999), the G2/M transcription factor forkhead box protein M1 (FOXM1) (Schimmel et al., 2014), PAX6 (Yan et al., 2010), nuclear hormone receptor ESR1 (Sentis et al., 2005) and EGR1 (Yu et al., 2009) (Table 2). Our early data reveal that SUMO1 modification of EGR1 contributes to EGR1-mediated PTEN transcriptional activation (Yu et al., 2009).

**FIGURE 4:**
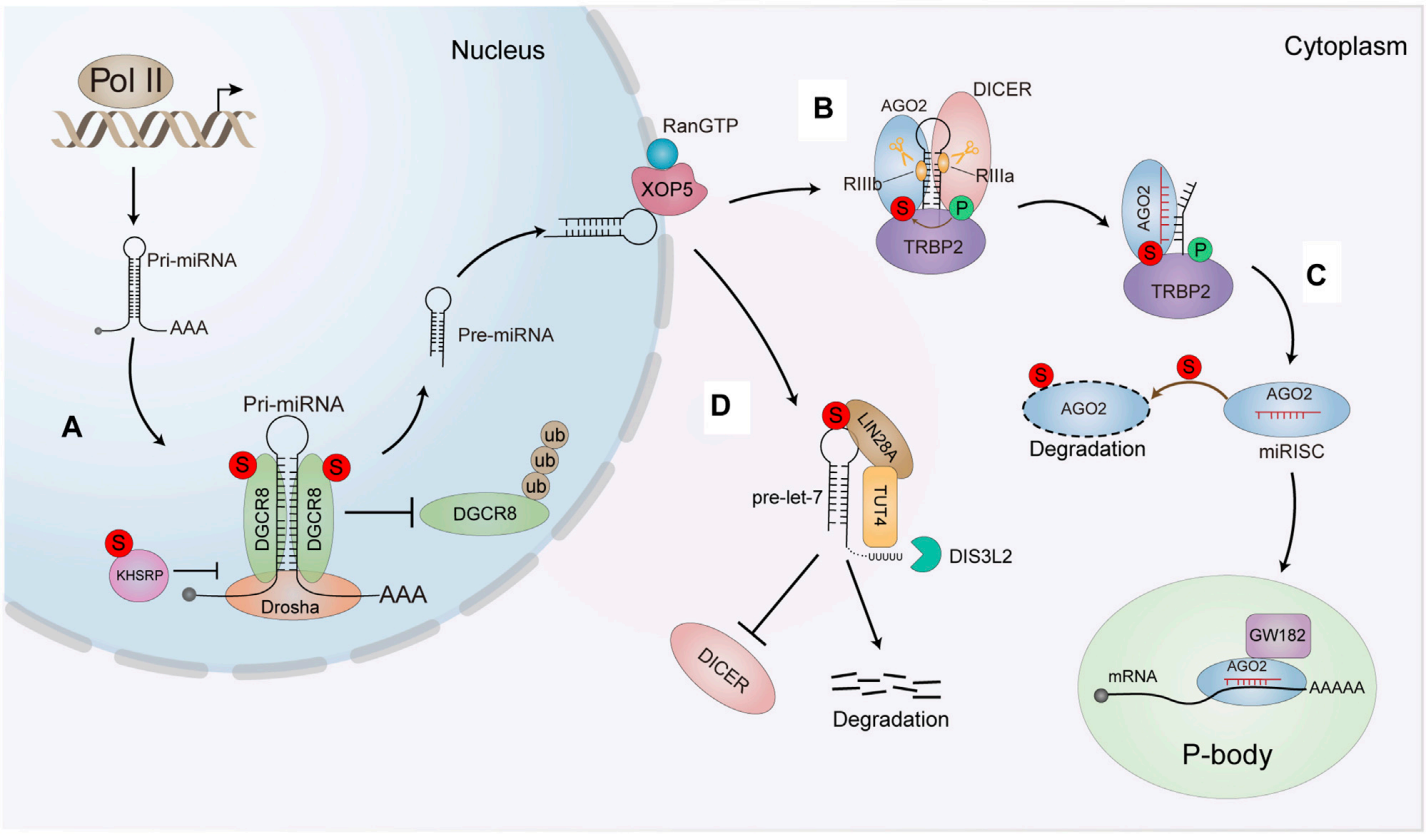
The effect of SUMO modification on the miRNA pathway. **(A)** OGCR8 SUMOyfation increases its protein stability by preventing the degradation *via* the ubiquitin proteasome pathway. The SUMOylation also enhances its affinity with pri-miRNA thus positively promoting the pri-miRNA direct recognition and repression of the targeted mRNA. SUMOylated KHSRP inhioits interaction with Dro-sha/DGCR8 and pri-miRNAs complex, sequentially downregulatlng a subset of miRNAS biogenesis. **(B)** SUMOylation of TARBP2 controls the efficiency of RNA-induced gene silencing by increasing its interaction with AGO2 and precursor miRNAs/siRNAs **(C)** SUMOytation enhances AGO2 turnover and antagonizes its stability. **(D)** SUMOylated of LIN28 A increases the binding affinity of LIN28 A and pre-let-7. Thereby leading to the degradation of pre•let-7 and reducing mature let-7 biogenesis. The intense interaction between LIN28 A and pre-let-7 can efficiently recruit TUT4 to urictylate pre-let-7 and block the processing of pre-let-7.

There are also some evidences show that SUMOylation of transcriptional machinery-related proteins can promote transcriptional activation. SUMO1 marks the chromatin on the promoters of many housekeeping genes, which encode translation factors and ribosomal subunit proteins, to promote the transcription of these specific RNAs (Liu et al., 2012). The C-terminal domain (CTD) of the largest subunit of RNA polymerase II (RNAP II) is important in coupling RNA processing and transcription (Corden and Patturajan, 1997; Bentley, 2014). The chromatin scaffold protein SAFB (scaffold related factor B) interacts with the CTD of RNAPII and a subset of serine-/arginine-rich RNA processing factors (SR proteins) to form a ‘transcriptosomal’ complex that couple the transcription and RNA processing (Nayler et al., 1998). SUMO1-modified SAFB1 can promote the binding of RNAPII to the promoters of ribosomal protein genes and pre-mRNA splicing (Liu et al., 2015) (Figure 2A; Figure 4).

The transcription profiling reveals that SUMOylation represses the global transcription by inhibiting transcriptional elongation. SUMOs and MYC oppositely control global gene expression by regulating the dynamic SUMOylation and deSUMOylation of CDK9. CDK9 is the catalytic subunit of the P-TEFb kinase and is essential for productive transcriptional elongation. SUMOylation of CDK9 represses global transcription, while MYC amplifies global transcription by antagonizing CDK9 SUMOylation (Yu et al., 2018) (Figure 2A). The oncogene MYC is part of a superfamily of genes encoding the most commonly activated oncoproteins in human cancers (Schaub et al., 2018). MYC is highly associated with cell proliferation, differentiation, survival and death (Bernard and Eilers, 2006; Bretones et al., 2015; Stine et al., 2015; Baluapuri et al., 2020). Dysregulation of MYC expression or activation has been linked to many human cancers (Dang, 2012; Lourenco et al., 2021). Many therapeutic agents targeting MYC are under development, among which the inhibitors of MYC transcriptional activity may be an attractive therapeutic intervention (Dhanasekaran et al., 2022). Considering that MYC amplifies gene expression by antagonizing CDK9 SUMOylation, modulation of CDK9 SUMOylation may be a feasible therapeutic approach given that there are currently no approved direct inhibitors of MYC.

In response to stresses, the transcription of housekeeping genes is usually downregulated in cells. Stress-induced SUMOylation of negative elongation factor (NELF) is required for global transcriptional repression by promoting the binding of NELF to the promoters, which impairs RNAPII elongation. NELF forms nuclear condensations in a dephosphorylation-dependent and SUMOylation-dependent manner under stressful conditions. Biomolecular condensation facilitates enhanced recruitment of NELF to promoters upon stress to drive transcriptional downregulation (Rawat et al., 2021) (Figure 2A). RNA polymerase II-associated factor 1 (PAF1)/pancreatic differentiation 2 (PD2) is a core subunit of the human PAF1 complex (PAF1C) that regulates the RNA polymerase II function during transcriptional elongation. SUMOylation of PAF1/PD2 predominantly presents in the nucleus and promotes its interaction with PML protein in response to radiation. PML functions in multiple aspects of cellular function including transcription. PML-NBs are strongly associated with the SUMOylation process, since SUMOylation of PML induces recruitment of other SIM-containing factors to these bodies (Zhong et al., 2000). Furthermore, inhibition of SUMOylation or PML reduces the cell growth and proliferation of pancreatic ductal adenocarcinoma (PDAC) cells (Rauth et al., 2021) (Figure 2A).

## mRNA processing

Most mRNA precursors (pre-mRNAs) undergo three processing steps: the 5′end is capped by addition of 7-methylguanosine; introns are removed and exons ligated by splicing; and the 3′end is created by an endonucleolytic cleavage followed by addition of a 100–300 nt long poly(A) tail. The evidence linking SUMOylation with the process of RNA capping, splicing and tailing is majorly based upon proteomic analysis (Richard et al., 2017). In the past decade, the affinity purification strategy based on tagged-SUMO peptides has led to the development of mass spectrometry (MS) identifying large-scale SUMOylated proteins, many of which are involved in RNA processing events such as capping, splicing, polyadenylation and mRNA export in mammals (Li et al., 2004; Manza et al., 2004; Gocke et al., 2005; Guo et al., 2005; Rosas-Acosta et al., 2005; Vertegaal et al., 2006; Blomster et al., 2009; Golebiowski et al., 2009; Matafora et al., 2009; Matic et al., 2010; Tatham et al., 2011; Becker et al., 2013; Schimmel et al., 2014; Tammsalu et al., 2014; Yang and Paschen, 2015). In yeast, a significant proportion (17%) of the SUMO-modified proteins identified is found to be involved in RNA-related processes (Denison et al., 2005). It is reported that enzymes in SUMO pathway are co-located in some nucleosomes, which is believed to be closely related to RNA processing. Nuclear speckle NS, for an example, plays a major role in regulating the availability of splicing factors at the transcription site since more than 50% of NS proteins are involved in transcription or splicing regulation. NS-associated processers are well regulated by SUMOylation because SUMO E2 Ubc9 (Ihara et al., 2008) and E3 ligases PIAS family (Tan et al., 2002) localize to NS. However, how SUMOylation cooperatively modulates the function of individual NS proteins remains to be revealed (Galganski et al., 2017). In addition, SUMO1 and Ubc9 are also localized to Cajal bodies, which are implicated in RNA-related metabolic processes (Navascues et al., 2008).

Pre-mRNA splicing is one of most important RNA processing catalyzed by the spliceosome. Spliceosome assembly occurs by the ordered interaction of five small nuclear ribonucleoprotein particles, termed U1, U2, U4, U5, and U6 small nuclear ribonucleoproteins (snRNPs). Each snRNP consists of a small nuclear RNA (snRNA) and a large set of associated proteins. In the earliest cross-intron spliceosomal complex, U1 and U2 snRNP bound to the intron to form A complex. Then, the activated spliceosome B Complex generated by the recruitment of U4/U6/U5 tri-snRNP. After numerous RNA and protein rearrangements, including the dissociation of the U1 and U4 snRNPs, and splicing by the DEAH-box RNA helicase PRP2, the catalytic C complex is finally yielded. The C complex catalyzes intron excision and ligation of the exons followed by spliceosome disassembly (Wilkinson et al., 2020). Several spliceosomal proteins are SUMOylated *in vitro* and in cultured cells, including components of U1, U2, U4/U6, U5 snRNPs, non-snRNPs, heterogeneous nuclear ribonucleoproteins (hnRNPs) as well as a wide range of other spliceosome-associated factors, suggesting that SUMOylation may play a role in multiple steps of pre-mRNA splicing (Pozzi et al., 2017; Chanarat and Mishra, 2018).

Serine/arginine (SR) proteins, such as members of the hnRNP family and many splicing factors, are highly SUMOylated in response to various stresses (Golebiowski et al., 2009; Psakhye and Jentsch, 2012). The SR protein SRSF1 (known as SF2/ASF) acts as a regulator of the SUMOylation pathway by interacting with Ubc9 to modulate the activity of E3 ligase PIAS1, thus promoting SUMOylation of specific substrates. SRSF1 is upregulated in varies cancer type (Ghigna et al., 2005; Karni et al., 2007; de Miguel et al., 2014; Anczukow et al., 2015) and controls alternative splicing of many tumor-related genes, which affect cell apoptosis (Olsson and Zhivotovsky, 2011; Anczukow et al., 2012; Gautrey and Tyson-Capper, 2012), proliferation and migration (Ghigna et al., 2005) thereby promoting tumorigenesis and cancer development. Knockdown of SRSF1 suppresses SUMO modification of splicing factors as well as tumor progression (Pelisch et al., 2010). The splicing factor U4/U6 small nuclear ribonucleoprotein PRP3, a component of the U4/U6 di-snRNP, is another SUMOylation substrates during splicing and its SUMOylation is regulated by SRSF1 (Li N et al., 2020). SUMOylation of PRP3 is required for U4/U6/U5 tri-snRNP formation and recruitment to activate the spliceosome. The SUMOylation-deficient mutant PRP3/2 KR do not affect PRP3 binding to U4 or U6 snRNAs, but prevented its interaction with the associated splicing factors U2-SF3A1 and U5-Snu114, suggesting that SUMOylation of PRP3 promotes U4/U6/U5 tri-snRNP formation to affect splicing (Pozzi et al., 2017) (Figure 2B).

SAFB, which interacts with the CTD of RNAPII and RNA processing proteins such as SR proteins as mentioned before, is identified as an SUMO1 substrate binding to promoters of highly expressing genes. SUMOylation of SAFB also increases splicing rates of mRNAs encoding ribosomal proteins, suggesting a role for SUMOylated-SAFB in coupling transcription and RNA processing (Liu et al., 2015). SAFB is also a key player in tumorigenesis. Overexpression of SAFB1 leads to growth inhibition of breast cancer cells (Townson et al., 2000), and it involved in apoptosis and the immune system as a transcriptional repressor regulating genes in breast cancer cells (Hammerich-Hille et al., 2010). However, the role of SAFB SUMOylation in cancer development is unclear. Except for factors directly involved in splicing, AKT is also involved in the splicing process as a key sensor that translates extracellular signals into changes in splicing patterns. AKT is modified by SUMO at K276 and K301 within its kinase domain, and its mutation (Akt E17 K) increases its SUMOylation levels in several human cancer types, enhancing its capacity to regulate fibronectin and Bcl-x alternative splicing patterns (Risso et al., 2013). In general, SUMOylation modulates the activity of splicing-related proteins to promote spliceosome assembly and improve splicing efficiency, which regulates alternative splicing of many tumor-associated genes to affect cancer progression, but more studies are needed to clarify the detailed mechanisms of SUMOylation of splicing related proteins in the regulation of tumorigenesis, so as to promote the improvement of cancer treatment strategies.

SUMOylation is also involved in regulating another key mRNA processing step, polyadenylation, which processes the 3′-end of nascent transcripts and requires several complexes including CFI (cleavage factor I), CFII, CPSF (cleavage and polyadenylation specificity factor) and CstF (cleavage stimulation factor) and additional protein factors (Shi et al., 2009). The 3′ends of pre-mRNAs are formed in a two-step process, with an endonucleolytic cleavage generating a 3′OH end followed by the synthesis of a poly(A) tail (Colgan and Manley, 1997; Proudfoot and O'Sullivan, 2002). For canonical 3′-end processing in mammals, the cleavage site is located between an upstream polyadenylation signal (PAS), most frequently an AAUAAA hexamer, and a GU-rich downstream element (DSE) (Sun et al., 2020). The multi-subunit CPSF and CstF define the poly(A) site by binding cooperatively to PAS and DSE, respectively. Cleavage factors I and II help in the complex assembly and in the first step, then Poly(A) polymerase (PAP) catalyzes the addition of poly(A) (Takagaki et al., 1989; Raabe et al., 1991). It has found that CPSF, PAP and assembly factor Symplekin are modified by SUMO2/3 (Richard et al., 2017) (Figure 2C). Blocking SUMOylation of CPSF and Simplekin affects pre-mRNA 3′complex assembly and activity and is lethal to cell viability (Vethantham et al., 2007; 2008). CPSFs have multiple roles in different tumor types. CPSF1 (Zhang et al., 2017a) and CPSF4 (Tang et al., 2016; Yi et al., 2016; Zhang et al., 2017a) promote cell proliferation in ovarian cancer and lung adenocarcinoma (LUAD), respectively, while CPSF2 inhibits invasion and cancer stem cell growth (Sung et al., 2015). However, the mechanism of CPSF SUMOylation in cancer remains unclear. PAP is highly SUMOylated in its C-terminal regulatory domain by strong binding with Ubc9, which is required for its correct nuclear localization and stability. *In vitro* polyadenylation assays show that SUMOylated PAP has lower poly(A) synthesis activity, indicating that SUMOylation of PAP inhibits polyadenylation activity to profoundly affect gene expression (Vethantham et al., 2008). Dysfunction of PAP activity has been shown to associated with diverse diseases. The increasing activity of PAP is correlated with malignancy in breast cancer and leukemia (Papamichail et al., 1983). Conversely, the inhibition of PAP activity is associated with apoptosis (Scorilas et al., 1998). Thus, regulation of PAP mediated by SUMOylation may have important role in cancer development and more studies are needed to investigate its mechanism.

## mRNA stability

SUMOylation regulates some of the key factors controlling RNA stability under cellular stresses. Nucleolin is a multifunctional DNA and RNA binding protein, closely associates with regulation of cell proliferation and growth, which is abundant in growing and cancerous cells (Grinstein et al., 2007). SUMOylation of nucleolin is required for maintaining its nuclear localization and promoting its activity in mediating *gadd45*α mRNA stability, which in turn increases apoptosis under arsenic exposure (Zhang et al., 2015). Therefore, it is likely that the SUMOylation of nucleolin has an important regulatory role in specific stress-induced mRNA stabilization.

N6-methyladenosine (m^6^A) is one of the most common modifications in mRNA, rRNA, tRNA, microRNA and long non-coding RNA (Alarcon et al., 2015). Human YTH domain family 2 (YTHDF2) selectively recognized m^6^A-RNAs to regulate degradation by recruiting the CCR4-NOT complex through a direct interaction between the YTHDF2 N-terminal region and the SH domain of the CNOT1 subunit (Du et al., 2016) (Figure 3A). We recently found that YTHDF2 can be modified by SUMO1 under hypoxic conditions, which enhances its binding affinity to m^6^A RNAs, leading to accelerated degradation of mRNAs. Subsequently, SUMOylation of YTHDF2 changes the gene expression profile, thus promoting cancer progression (Hou et al., 2021).

In response to various environmental stresses, stress granules (SGs) rapidly form thus transiently storing mRNAs and RNA-binding proteins (RBPs) to limit protein synthesis. SGs are associated with various diseases including cancers, viral infections and neurodegenerative diseases (Ivanov et al., 2019). It has been demonstrated that SUMO-conjugating enzymes are recruited to SGs and SUMOylation regulates SG formation and disassembly (Keiten-Schmitz et al., 2020; Marmor-Kollet et al., 2020). As reported by several groups, the SUMOylation level of RBPs is regulated under stress conditions, however how SG regulates RNA metabolism remains unclear.

## RNA modification and RNA editing

SUMOylation has been implicated in RNA modifications. m^6^A is the most prevalent internal RNA modification in eukaryotes. It is a dynamic and reversible modification that occurs primarily in 3′untranslated regions (3′UTRs) and near the stop codons (Meyer et al., 2012). The methyltransferase like 3 (METTL3) is a key component of the large N6-adenosine-methyltransferase complex in mammalian. Our laboratory found that METTL3 is modified by SUMO1, which does not affect its stability, subcellular localization, or interaction with methyltransferase complex METTL14/WTAP, but significantly inhibits its m^6^A methyltransferase activity. SUMOylation of METTL3 enhances tumor growth in human non-small cell lung carcinoma cell line H1299 by decreasing the m^6^A level in mRNAs and subsequently changing gene expression profile (Du et al., 2018). XU et al confirmed SUMOylation of METTL3 can regulate hepatocellular carcinoma (HCC) progression *via* controlling *Snail* mRNA homeostasis in a m^6^A methyltransferase activity-dependent manner (Xu et al., 2020). ROS induces a global increase in mRNA m^6^A *via* inhibition of the m^6^A demethylase ALKBH5. Mechanistically, ROS promotes ALKBH5 SUMOylation through activating ERK/JNK signaling, leading to inhibition of ALKBH5 m^6^A demethylase activity by blocking substrate accessibility. Increased global mRNA m^6^A levels lead to the rapid and efficient induction of thousands of genes involved in a variety of biological processes including DNA damage repair (Yu et al., 2021) (Figure 3A). Methylases and demethylases of m^6^A can affect the complexity of cancer progression (Zhang et al., 2017b; Weng et al., 2018; Huang et al., 2019; Li J et al., 2020), thus SUMOylation of remaining enzymes should be investigated for potential clinical application of cancer treatment.

5-methylcytosine (m5C) is another important posttranscriptional modification of RNA. The modification m5C in mRNAs is mainly catalyzed by the RNA methyltransferase NSUN2 and specifically recognized by the mRNA export adaptor ALYREF (Yang et al., 2017). NSUN2 is highly expressed in various tumors but extremely low in most normal tissues (Okamoto et al., 2012). NSUN2 is modified by SUMO2/3 stabilizing NSUN2 and mediating its nuclear transport, which regulates the m5C modification levels of mRNAs that are involved in GC progression (Hu et al., 2021) (Figure 3B).

ADAR1, an RNA-editing enzyme also undergo SUMOylation to regulate RNA metabolism. ADAR1 binds to double-stranded RNA and converts adenosine to inosine, resulting in changes in the amino acid coding that alter protein sequence/function (Baker and Slack, 2022). ADAR1 is commonly overexpressed in a growing number of cancer types, and its oncogenic role in cancers is attributed to multiple mechanisms. It has been found that ADAR1 can be modified by SUMO1. Although SUMOylation does not affect ADAR1 localization in the nucleolus, it appears to inhibit its RNA editing activity (Desterro et al., 2005) (Figure 3C).

## Non-coding RNA metabolism

Non-coding RNAs (ncRNAs) are a class of RNA molecules that do not encode proteins but can function except mRNA, tRNA and rRNA. miRNAs are small non-coding regulatory RNAs ranging from 20–25 nucleotides, which binding to partially complementary sequences within the 3′-untranslated regions of target mRNAs inducing translational repression or mRNA degradation (Treiber et al., 2019). miRNAs are extensively deregulated in human cancers, as miRNA expression is globally suppressed in tumor cells compared to normal cells (Lu et al., 2005). miRNA biogenesis is strictly controlled at several levels, such as transcription, processing, itself modification and decay and is closely related to tumors (Ha and Kim, 2014). Aberrant miRNA biogenesis in cancer occurs at different steps during miRNA maturation, and more importantly, key proteins of miRNA biogenesis are post-transnationally modified by SUMOs to regulate miRNA biogenesis and functions **(** Table 3 **)**.

**TABLE 3:** SUMOylation affects miRNA biogenesis and functions in cancer.

Protein	SUMOylation mechanism	SUMO regulation in miRNA biogenesis	SUMOylation role in tumor	References
DGCR8	Promote by phosphorylation; enhances protein stability by blocking ubiquitination; increases affinity with pri-miRNA	Increases miRNA biogenesis and the microprocessor activity	Promote tumorigenesis and cancer progression	Zhu et al. (2015), Zhu et al. (2016)
KHSRP	Promotes cytoplasmic localization; interferes interaction with pri-miRNAs	Inhibits the biogenesis of TL-G-Rich miRNAs	Promote tumorigenesis and cancer progression	Yuan et al. (2017)
TARBP2	Stabilizes TARBP2; promotes binding with pre-miRNAs; stabilization of AGO2 by increasing the binding between them	Efficiency miRNA-inducing gene silencing	Suppress tumor progression	Chen et al. (2015)
AGO2	Negatively regulates AGO2 turnover	Does not alter RNA interference		Sahin et al. (2014)
LIN28 A	Increases its binding to pre-let-7; promotes pre-let-7 uridylation and inhibits pre-let-7 processing	Enhances inhibition of let-7 biogenesis	Weaken oncogenic capacity	Dou et al. (2020)

Primary miRNAs (pri-miRNAs) are typically transcribed by RNA polymerase II in the nucleus (Krol et al., 2010), and then pri-miRNAs are cleaved into approximately 70 nucleotide stem-loop structures by the nuclear RNase III-type enzyme DROSHA, in a complex with its co-factor DGCR8 (DiGeorge syndrome critical region 8 homolog), which is essential for pri-miRNA processing by cleaving pri-miRNA into pre-miRNA in the nucleus. We first reported that DGCR8 can be modified by SUMO1 at K259 and K707. K707-SUMOylation of DGCR8 enhances protein stability by preventing degradation by the ubiquitin-proteasome pathway (Zhu et al., 2015), whereas K259-SUMOylation of DGCR8 is critical for its nuclear localization for its normal miRNA processing functions (Zhu et al., 2016). SUMOylation of DGCR8 at K707 does not alter its association with Drosha and miRNA biosynthesis. However, K707-SUMOylation affects its affinity for pri-miRNA to control the direct function of pri-miRNAs in recognizing and repressing target mRNAs (Zhu et al., 2015). SUMOylation of DGCR8 is apparently related to the function of DGCR8 in regulating tumorigenesis and cell migration in lung adenocarcinoma (Figure 4A **).** The hnRNP K homology (KH)-type splicing regulatory protein (KHSRP) is one major component of the DROSHA complex. KHSRP can promote the biogenesis of miRNAs, such as let-7 family, whose pri-miRNAs harboring short G-rich stretches in their terminal loops (TL). SUMO1 modification of KHSRP at K87 can be induced upon the microenvironmental hypoxia while reduced by the treatment with growth factors. SUMOylation of KHSRP probably increases its translocation from the nucleus to the cytoplasm, thus inhibiting its interaction with the pri-miRNA/DROSHA-DGCR8 complex, therefore SUMOylation of KHSRP results in the downregulation of TL-G-Rich miRNAs such as let-7 family, which is linked to tumorigenesis and cancer progression (Figure 4A **)** (Yuan et al., 2017).

In the cytoplasm, pre-miRNAs are cleaved into double-stranded RNAs (dsRNAs) by another RNase III-type enzyme, DICER with its co-factors TRBP (TAR RNA-binding protein 2, also known as TARBP2), resulting in an RNA duplex of ∼22 nucleotides. The short RNA duplex is bound by an AGO (Argonaute) protein, a component of a multi-subunit complex termed miRISC (miRNA-induced silencing complex) (Iwakawa and Tomari, 2022). Finally, miRISC mediates the recognition of targeted mRNAs by guiding miRNA to their specific mRNA targets through base pairing (Zealy et al., 2017). Interestingly, we found that SUMOylation of TARBP2 regulates the efficiency miRNA/short interfering RNA (siRNA) (Chen et al., 2015). TARBP2 is SUMOylated at K52, which can be enhanced by its phosphorylation. SUMOylation of TARBP2 significantly promotes its binding with pre-miRNAs to facilitating the efficiency miRNA-inducing gene silencing. SUMOylated TARBP2 also represses its ubiquitination and stabilizes AGO2 by increasing the binding with AGO2 *via* SIMs of AGO2. SUMOylation of TARBP2 are implicated in suppression of tumor growth and tumor cell migration (Figure 4B) (Chen et al., 2015). Moreover, AGO2 can be modified by SUMO1 and SUMO2/3 leading to reduce its stability (Figure 4C) (Sahin et al., 2014). However, our unpublished data suggested that SUMOylation of AGO2 does not affect its stability, but crosstalk with phosphorylation together modulates miRNA loading to AGO2, which affects the gene silencing activity.

In addition to key proteins in these classical miRNA biogenesis pathways, other proteins also undergo SUMO modification to affect miRNA metabolism. Let-7 family members (let-7 s) are known as important tumor suppressors, which are downregulated in multiple cancers, and associated with increased proliferation and invasion of cancer cells. LIN28 A is a conserved RNA-binding protein that inhibits processing of pre-let-7 miRNAs, thus promoting cancer progression. Our lab showed that LIN28 A is SUMOylated at K15, which is increased by hypoxia but reduced by chemotherapy drugs such as Cisplatin and Paclitaxel. SUMOylation of LIN28 A increases its binding affinity with the pre-let-7 and recruits of terminal uridylyltransferase TUT4 to block processing of pre-let-7. Subsequently, SUMOylation of LIN28 A exacerbates its inhibition of let-7 maturation, thereby reducing mature let-7 production. These effects promote cancer cell proliferation, migration, invasion and tumor growth in DU145 and T47D cell lines (Figure 4D) (Dou et al., 2020).

PIWI interacting RNAs (piRNAs) and long non-coding RNAs (lncRNAs) are also influenced by SUMOylation. Several studies in *Drosophila* have demonstrated the role of SUMOylation in primary piRNA biogenesis (Muerdter et al., 2013) and piRNA-guided transcriptional silencing and heterochromatin formation (Mugat et al., 2020; Ninova et al., 2020; Andreev et al., 2022; ElMaghraby et al., 2022). Moreover in *C. elegans*, PIE-1 is itself modified by SUMO and acts as a SUMO E3 together with the conserved SUMO-E3 GEI-17/PAIS1 to promote piRNA-dependent silencing. PIE-1 SUMOylation promotes SUMOylation of HDA-1 and the assembly of a MEP-1/Mi-2/HDA-1 chromatin remodeling complex in the adult germline to promote fertility and embryonic development (Kim et al., 2021). However, there is relatively limited study of SUMOylation on the function of piRNAs in cancers.

A growing body of evidence has shown that many lncRNAs contribute to regulate the SUMOylation of some proteins, leading to cancer development and progression. LncRNA myosin heavy chain associated RNA transcript (MHRT) can regulate the SUMOylation levels of NAD-dependent protein deacetylase sirtuin-1 (SIRT1), peroxisome proliferator-activated receptor γ coactivator-1 α (PGC-1α)/peroxisome proliferator-activated receptor-α (PPARα), specificity protein 1 (SP1)/HDAC4 in cardiac hypertrophy (Liu et al., 2022). LncRNA *ELNAT1* promotes lymphangiogenesis and lymph node (LN) metastasis in bladder cancer (BCa) cell lines by inducing Ubc9 overexpression to catalyze SUMOylation of hnRNPA1 (Chen et al., 2021). LncRNA small nucleolar RNA host gene 1 (SNHG1) enhanced SUMOylation of Bhlhe40 protein by facilitating the binding of SUMO E3 ligase PIAS3 to Bhlhe40, resulting in increased nuclear translocation of Bhlhe40 (Li S et al., 2022). LncRNA SDCBP2-AS1 binds to hnRNP K to repress its SUMOylation, which facilitates the ubiquitination of hnRNP K and β-catenin, thereby promoting the degradation of β-catenin and suppressing tumorigenesis and metastasis in gastric cancer (Han et al., 2022). Oncogene glucose transporter 1 (GLUT1) associated lncRNA (GAL) interacts with GLUT1 protein, which increases GLUT1 SUMOylation and inhibits the ubiquitin proteasome system on the GLUT1 protein, thus promoting colorectal cancer cell migration and invasion (Li B et al., 2022). LncRNA rhabdomyosarcoma 2 associated transcript (RMST) enhances FUS SUMOylation, which contributes to the interaction between FUS and hnRNP D to suppress glioblastoma cell mitophagy (Liu C. et al., 2020). LncRNA RP11-214F16.8 decreases the protein level of tumor suppressor NISCH by replacing SUMOylation with ubiquitination, which drives breast cancer (Lv and Zhang, 2022). LncRNA p53-stabilizing and activating RNA (*PSTAR*) can bind to hnRNP K and enhance its SUMOylation, thereby strengthening the interaction between hnRNP K and p53, ultimately resulting in the accumulation and transactivation of p53 to suppress hepatocellular carcinoma (Qin et al., 2020). Interestingly, c-Myc promotes p53 polyubiquitination and turnover by reducing p53 SUMOylation through c-Myc-inducible LncRNA inactivating P53 (MILIP), driving cancer pathogenesis (Feng et al., 2020). In addition, lncRNA TINCR can encode an evolutionary conserved ubiquitin-like protein (UBL) named pTINCR. pTINCR increases CDC42 SUMOylation and promotes its activation, leading to epithelial differentiation and tumor suppression, which acts as a tumor suppressor in epithelial cancers (Boix et al., 2022). These findings suggest lncRNA regulating SUMOylation can be as a new potential direction for the cancer treatment strategies.

## Concluding remarks

In the past decade, SUMOylation research has found a large set of SUMO target proteins, which regulate the different stages of RNA metabolism. In this article, we summarize the regulatory roles of SUMOylation in RNA metabolism in cancer cells. A tight control of mRNA metabolism is crucial to allow cells in response to physiological stresses. SUMO proteome with key proteins/enzymes of mRNA transcription, splicing or nuclear complex is subject to spatio-temporal regulation and cellular stress remodeling to control mRNA metabolism. Since most of SUMOylation substrate proteins are localized in the nucleus, the effect of SUMOylation is mainly to inhibit the global transcription activity. When SUMOylation sites are located within the inhibitory or repressive domains, mutations of these sites can dramatically enhance the transcriptional activity.

MiRNA biogenesis is closely regulated by SUMOylation, thus affecting tumorigenesis and cancer progression. SUMOylation has no uniform effect on miRNA biogenesis process and miRNA-induced RNA silencing efficiency. Considering that the activity and abundance of core proteins and their partners in different stages of miRNA biogenesis are not consistent in different cancers, there is no comprehensive understanding and definite conclusion on how SUMO specifically affects miRNA metabolism in specific types of cancer. Moreover, a lot of lncRNAs mediate SUMOylation of target proteins, which plays complex and precise regulatory roles in cancer progression. However, the role of SUMOylation in the transcription, processing, stability and functions of lncRNAs is limited and unclear. Finally, understanding the impact of SUMOylation on RNA metabolism may provide new therapeutic strategies for cancer treatment.
